# Oxytocin, GABA, and TRPV1, the Analgesic Triad?

**DOI:** 10.3389/fnmol.2018.00398

**Published:** 2018-11-13

**Authors:** Abimael Gonzalez-Hernandez, Alexandre Charlet

**Affiliations:** ^1^Departamento de Neurobiología del Desarrollo y Neurofisiología, Instituto de Neurobiología, Universidad Nacional Autónoma de México, Queretaro, Mexico; ^2^Centre National de la Recherche Scientifique, Institut des Neurosciences Cellulaires et Intégratives, Strasbourg, France

**Keywords:** oxytocin, pain, nociception, TRPV1, GABA

The hypothalamic non-apeptide oxytocin (OT) has several physiological functions, ranging from lactation to social attachment (Gimpl and Fahrenholz, 2001). More recently, a collection of evidence revealed that OT inhibits pain transmission at peripheral (Juif et al., 2013; de Araujo et al., 2014; Tzabazis et al., 2016; González-Hernández et al., 2017), spinal, and supraspinal levels (Eliava et al., 2016; Poisbeau et al., 2018). These data support the prospect that spinal OT could be translated into clinical pain practice (Rash et al., 2014; Eisenach et al., 2015; Condés-Lara et al., 2016). While the receptor(s) and intracellular mechanisms responsible for the OT-induced analgesia are under scrutiny, the main dogma relies on the activation of the OT receptor (OTR).

Indeed, *ex vivo* studies using capsaicin-sensitive dorsal root ganglia (DRG) neurons, showed that OT induces membrane hyperpolarization and consequently inhibits the nociceptive transmission by Ca^2+^-dependent mechanisms (Hobo et al., 2012). Additionally, *in vivo* electrophysiological recordings of the second-order wide-dynamic-range (WDR) neurons and *ex vivo* patch-clamp as well as behavioral analyses, suggest that OT inhibits the nociceptive input either directly (Eliava et al., 2016), by the enhancement of the GABAergic transmission (Rojas-Piloni et al., 2007; Breton et al., 2008), or by inducing neurosteroids synthesis (Juif et al., 2013). In all cases, the OTR was involved. However, the contribution of the vasopressin V_1A_ receptor (V_1A_R), for which OT has a physiologically relevant affinity, cannot be ruled out (Schorscher-Petcu et al., 2010; González-Hernández et al., 2014; Manzano-García et al., 2018). Regardless, these findings point to the relevance of GABAergic transmission in the OT-induced analgesia (Figure 1).

**Figure 1:**
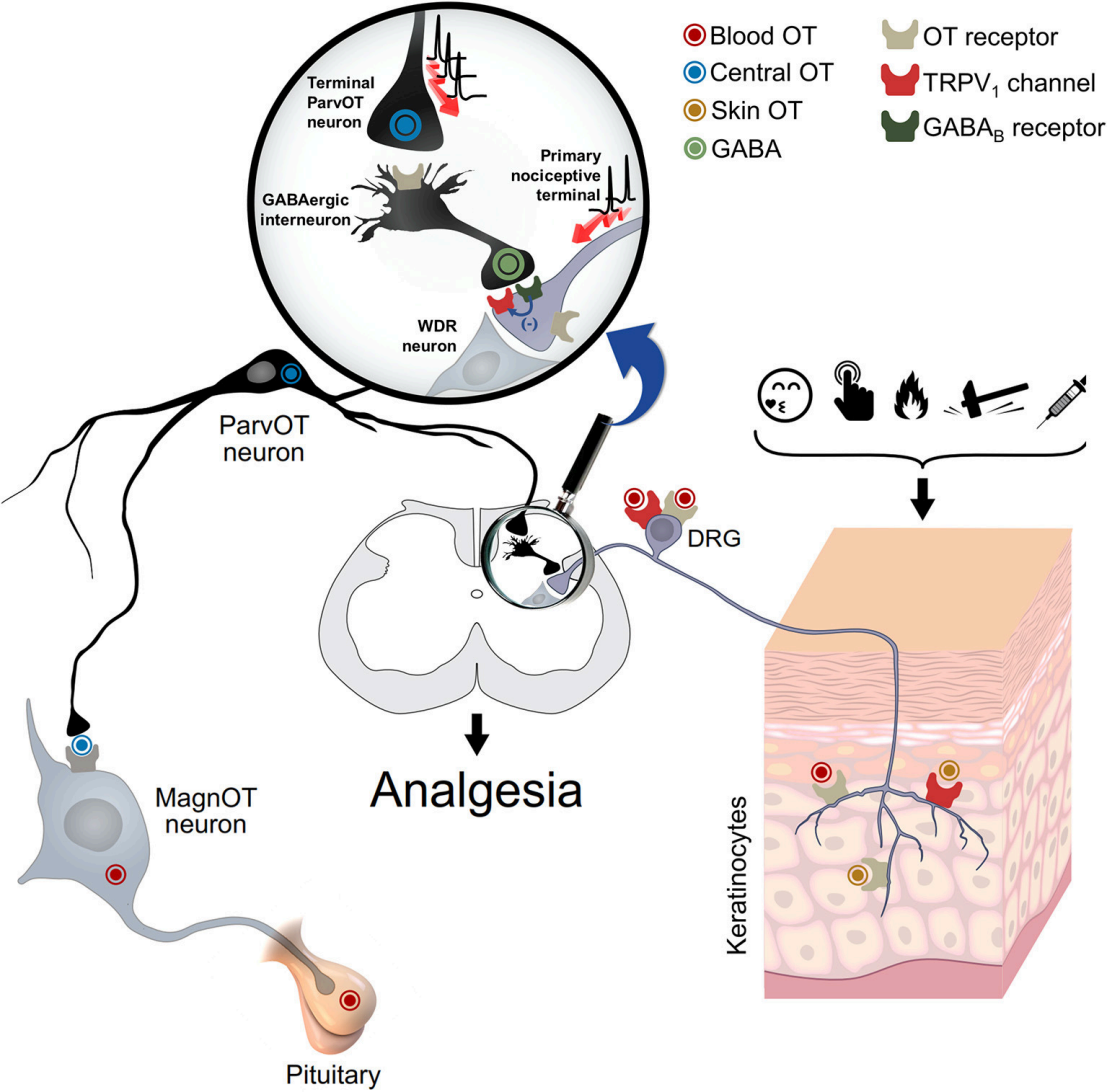
Oxytocin and spinal and peripheral analgesia. This schema depicts the proposed mechanism for the oxytocin-mediated peripheral analgesia. Oxytocin could act via either OTR and/or TRPV1 through a three-way path: (i) spinal cord central release, to inhibit WDR neuron activity either directly or via activation of GABAergic neurons; (ii) blood release, through pituitary, to inhibit either the cell body or peripheral nociceptive fibers of DRG neurons; and (iii) skin release, to directly inhibit the excitation of nociceptive fibers. WDR: wide-dynamic-range; DRG: dorsal root ganglia. Adapted from Grinevich and Charlet (2017).

In this context, two recent studies (Nersesyan et al., 2017; Sun et al., 2018) add a new layer to the analgesic mechanisms of the OT, suggesting that apart from the role of OTR and its downstream mechanism, this peptide also interacts (directly or indirectly) with the transient receptor potential vanilloid type1 (TRPV1) at peripheral and spinal levels. Certainly, since this channel physiologically functions as a detector of noxious stimuli (Caterina et al., 2000) and appears to be crucial for the transition from acute to chronic pain (Choi et al., 2016), there is a strong motivation to develop molecules modulating TRPV1 to treat neuropathic pain.

Nersesyan et al. was first to show that aversive behaviors induced by an intracutaneous (i.c.) injection of capsaicin into an animal hindpaw, were reduced in animals concomitantly treated with i.c. OT. One can therefore propose that the OT effect is a result of OTR activation at the peripheral nerve endings (Gong et al., 2015; González-Hernández et al., 2017; Figure 1), challenging the authors hypotheses of direct OT interaction with the TRPV1 channel. Indeed, since OT could modulate the influx of extracellular Ca^2+^, the role of voltage-gated or ligand-gated channels may play a role, as previously suggested (Sanborn et al., 1998). Based on this reasoning, they showed that OT at μM concentration elicits a Ca^2+^ influx through TRPV1 expressed in HEK293 cells. Furthermore, the proposed hypothesis was strengthened considering that both the TRPV1 antagonist capsazepine and a TRPV1-specific small interfering RNA (siRNA) abolished the OT-induced Ca^2+^ influx. These results were confirmed by replicating both the nociceptive DRG neurons isolated from wild-type animals and the modest OT-induced analgesia observed in TRPV1 knock-out mice. Further electrophysiological, planar lipid bilayer, and *in silico* experiments showed that OT acts as a partial agonist of TRPV1 channels and induces a strong desensitization of these channels, under nociceptive stimulation. This pioneering study strongly suggests that at peripheral level, OT-induced analgesia passes at least in part, through a direct TRPV1 interaction.

To further explore if OT-induced analgesia in neuropathic pain may occur via TRPV1, Sun et al. first confirmed that intrathecal OT diminishes the aversive behavior in neuropathic animals (Miranda-Cárdenas et al., 2006). Interestingly, the cerebrospinal fluid's OT concentration was increased in accordance with the spinal OT increase, measured after a peripheral inflammatory sensitization (Juif et al., 2013). Furthermore, while the spontaneous excitatory postsynaptic currents (sEPSC) recorded in superficial layers of the spinal cord, were not altered by OT, it prevented the capsaicin-induced increase of sEPSC, an effect blocked by the GABA_B_ receptor antagonist saclofen, suggesting a pre-synaptic effect. OT therefore enhanced the spontaneous inhibitory postsynaptic currents (sIPSC) mediated by GABA. These data suggest that OT recruits GABAergic interneurons, which in turn, activate GABA_B_ receptors and consequently inhibit the capsaicin-sensitive neurons. Interestingly, TRPV1 and GABA_B_ receptors were found to be coexpressed in primary nociceptive neurons. Additionally, the TRPV1 spinal cord expression was enhanced in neuropathic rats, an up-expression corrected by intrathecal OT treatment. Accordingly, the OT-induced analgesia was partially reduced in TRPV1 knockout mice. Like the proposal of Nersesyan et al., this study points out that OT-induced analgesia may rely on both GABA release and modulation of TRPV1 activity.

In the past decades, OT has slowly emerged as an important mediator of endogenous analgesia (Poisbeau et al., 2018). Thus, the relevance of oxytocinergic mechanisms implicated in the modulation of nociceptive transmission is under intense scrutiny. These two studies highlight a novel and interesting mechanistic explanation about how OT modulates the nociceptive transmission at the peripheral and spinal levels, under physiological and pathological pain conditions. Several research groups have reported GABAergic enhancement induced by OT at the spinal level, however, Sun et al. demonstrate that, this GABA facilitation engages the function of postsynaptic GABA_B_ receptors, which in turn modulate the TRPV1 function during neuropathic pain. This finding is in line with the observation that GABA_B_ receptors revert the TRPV1 sensitization by noncanonical signaling under pathological pain (Hanack et al., 2015). Additionally, the discovery by Nersesyan et al., that OT could act as an allosteric modulator of TRPV1, highlights the multifaceted actions of OT as a neuromodulator. Certainly, the finding that OT partially blocked the peripheral capsaicin-induced nocifensive behavior, could partly explain why blocking OTR at the peripheral level, only partially reverts the OT antinociceptive effect (González-Hernández et al., 2017).

An intriguing point in Sun et al. study is how exactly, at the spinal level, OT engages the intracellular machinery to modulate the TRPV1 expression. At least two mechanisms can be proposed. The first might be a direct action of OT on TRPV1, as suggested by Nersesyan et al. The second might be that the OT-induced activation of OTR, leads to an intracellular cascade capable of modulating the expression or epigenetic modifications of TRPV1, as suggested by Sun et al. It remains unclear however, how exactly OT modulates the TRPV1 functions and whether it remains true at physiological concentrations. Indeed, while Nersesyan et al. clearly show a direct interaction between OT and TRPV1, neither them nor Sun et al. were able to reveal a strong, behaviorally relevant, OT-mediated TRPV1 modulation. Since OTR was recently involved in functional heterodimers, such as vasopressin or dopamine receptors (Terrillon et al., 2003; Meyer-Lindenberg et al., 2011; De la Mora et al., 2016), the hypothesis of a functional heterodimer formation between OTR and TRPV might be interesting to explore further.

Finally, although the sources of OT and its spinal effects are neuronal and seem to play a role in endogenous analgesia (Eliava et al., 2016), it is more unclear at the peripheral level. Nersesyan et al. suggest that the OT which stimulates TRPV1 is neuronal, we need to keep in mind that other sources could explain the findings reported, such as keratinocytes (Denda et al., 2012), which may release OT upon nociceptive stimulation or self-soothing and consequently induce pain relief (Grinevich and Charlet, 2017; Walker et al., 2017; Figure 1). Certainly, the pleiotropic nature of this peptide, to modulate pain transmission, indicates that differential routes are followed.

In conclusion, a collection of evidence, briefly explained here, suggests that a triad between OT, GABA, and TRPV1 does exist and sheds a light on new mechanisms involved in OT-induced analgesia, which ultimately is much more complex than initially expected.

## Author contributions

All authors listed have made a substantial, direct and intellectual contribution to the work, and approved it for publication.

### Conflict of interest statement

The authors declare that the research was conducted in the absence of any commercial or financial relationships that could be construed as a potential conflict of interest.
